# Translation, cultural adaptation, and psychometric validation of the Arabic version of the university of Washington quality-of-life questionnaire in Jordanian head-and-neck cancer patients

**DOI:** 10.3389/fonc.2026.1866531

**Published:** 2026-07-15

**Authors:** Da’ad Abdel-Hay, Osama Abdelhay, Ra’ad Al Sunna, Mutaz A. Al Tamimi

**Affiliations:** 1 Department of Surgery, King Hussein Cancer Centre, Queen Rania Street, Amman, Jordan; 2 Department of Data Science and Artificial Intelligence, Princess Sumaya University of Technology, Khalil Saket Street, Amman, Jordan; 3 Department of Nursing, King Hussein Cancer Centre, Queen Rania Street, Amman, Jordan

**Keywords:** Arabic translation, cultural adaptation, head and neck cancer, psychometric validation, quality of life, UW-QoL v4

## Abstract

**Purpose:**

The University of Washington Quality of Life Questionnaire (UW-QoL v4) is widely used to assess health-related quality of life (HR-QoL) in patients with head and neck cancer (HNC). This study aimed to translate, culturally adapt, and validate an Arabic version of the UW-QoL v4 for use with Jordanian HNC patients.

**Methods:**

A two-phase cross-sectional study was conducted at King Hussein Cancer Centre. Phase one included forward and back translation by bilingual experts, expert panel review, and cognitive testing with Arabic-speaking participants to ensure linguistic and conceptual equivalence. Phase one participants were not included in phase two analyses. Phase two assessed the psychometric properties of the final Arabic version in 98 HNC patients from 118 consented participants. Internal consistency, two-item Spearman-Brown reliability, test-retest reliability using intraclass correlation coefficients, measurement error, exploratory structural validity using principal component analysis, floor/ceiling effects, and selected construct-validity hypotheses were evaluated. Statistical analyses followed COSMIN-informed recommendations and were conducted in R.

**Results:**

The Arabic UW-QoL v4 demonstrated good internal consistency: Cronbach’s alpha = 0.89 for the total 12-item scale, Cronbach’s alpha = 0.86 for the 10-item physical-function subscale, and Spearman-Brown = 0.86 for the two-item socio-emotional subscale. Test-retest reliability was excellent for the total score, ICC(A,1) = 0.95. Exploratory PCA supported a two-component solution, with the first two eigenvalues equal to 5.67 and 1.06. Floor effects ranged from 4.1% to 14.3%, whereas ceiling effects ranged from 26.5% to 50.0%. Associations between domain scores and patient-identified problems were in the expected negative direction (r = -0.45 to -0.30). Known-groups validity showed higher total scores in the early primary tumor stage (T1-2) than in the advanced primary tumor stage (T3-4), Mann-Whitney U test, p = 0.0025; rank-biserial correlation = 0.49.

**Conclusion:**

The Arabic version of the UW-QoL v4 is a reliable and culturally appropriate tool for assessing quality of life among Jordanian HNC patients. The findings provide initial evidence of structural and construct validity; further studies using externally validated comparators, broader clinical subgroups, and longitudinal designs are recommended.

## Introduction

Assessing the general quality of life (QoL) is a key method for evaluating various aspects of our lives, including psychosocial, health, and functional factors. When evaluating the QoL of individuals diagnosed with serious illnesses like cancer, it becomes crucial to determine how the disease might impact various aspects of a patient’s life (1). In recent years, the QoL for head and neck cancer (HNC) patients has gained importance as an outcome measure due to the anatomical characteristics of the HNC and the side effects of treatment. These factors can adversely affect the cosmetic appearance and lead to varying speech, swallowing, and voice dysfunction (2). Assessing health-related quality of life (HR-QoL) among cancer patients in primary care is crucial for informing treatment strategies and reducing the overall burden of the disease (3). HR-QoL can be measured through various questionnaires (4), with specific tools designed to assess the quality of life in patients with HNC (4, 5). These specialized questionnaires provide tailored insights that help to guide patient care and improve outcomes. One of the most widely used questionnaires for assessing QoL in HNC patients is the University of Washington Quality of Life questionnaire (UW-QoL). This tool is recognized globally for its ability to comprehensively evaluate the impact of HNC and its treatments on a patient’s quality of life (4, 6). The UW-QoL is an English-language assessment tool that has been shown to be valid and reliable for evaluating quality of life among English-speaking HNC patients (2, 4, 7).

Despite its widespread use and utility in HNC research, the UW-QOL questionnaire has known limitations. First, the instrument’s focus on specific functional and physical symptoms may underrepresent broader psychological and psychosocial domains, which are important aspects of overall quality of life in cancer survivors (8). Second, some experts caution that the UW-QOL may not fully capture the complexity of overall quality of life, particularly because its scoring assumes equal weighting of domains and uses single-item measures that may lack depth and sensitivity (8). Third, certain domains may be differentially relevant depending on treatment type, potentially affecting interpretation across heterogeneous patient groups (8). Finally, while the UW-QOL is brief and practical for clinical settings, it may not adequately address longer-term survivorship concerns beyond physical symptoms (9).

These recognized limitations prompted the development of a revised version, the University of Washington Quality of Life questionnaire version 4 (UW-QOL v4), which aims to address gaps in domain coverage, improve sensitivity to treatment- and disease-specific issues, and enhance the tool’s usability for patients and clinicians. The UW-QOL v4 is concise, easy to administer, and structured into four sections: 12 questions assessing specific domains of physical and functional well-being; a ranking of importance across 13 domains; three questions evaluating overall quality of life; and a free-text section for additional comments. To ensure its accuracy and applicability across different cultural contexts, the UW-QOL v4 has been translated and validated in several languages, including Brazilian Portuguese, Hindi, Spanish, and Turkish, with studies confirming its reliability and validity in these populations (8). Translating and adapting the UW-QoL v4 for these languages involved testing its validity and reliability in their respective countries (2).

Because of the varied dialects within the Arabic language and the lack of previous applications of this questionnaire in Jordan for HNC patients, the objectives of this study were to translate the original English version of the UW-QOL v4 into formal Arabic and to assess its validity and reliability for Arabic-speaking HNC patients in Jordan at King Hussein Cancer Centre (KHCC).

## Materials and methods

The current cross-sectional study was conducted in accordance with the Declaration of Helsinki. Ethical approval was obtained from the King Hussein Cancer Center KHCC. Institutional Review Board (IRB approval number: 22 KHCC 165). All methods were performed in accordance with relevant guidelines and regulations. Written informed consent was obtained from all individual participants included in the study.

The study was conducted in two phases. The first phase involved linguistic and cultural validation of the UW-QOL-V4 from English into Arabic. In contrast, the second phase focused on assessing the statistical validity and reliability of the Arabic version of the UW-QoL-V4.

### First phase: linguistic and cultural validation

Linguistic validation aims to produce an Arabic translation that is conceptually equivalent to the original while ensuring clarity and ease of understanding (10). This phase consists of three steps that follow internationally recognized guidelines (10–12). The initial step, forward translation, was performed by two native Arabic-speaking healthcare professionals with medical expertise. One was a senior speech-language pathologist with a master’s degree who had worked with head and neck patients for over 10 years (D.A.); the other was an ENT consultant (R.S.). Both are bilingual and independently translated the English version of the UW-QoL v4 into Arabic, resulting in two separate translations. Next, a native Arabic-speaking linguistics professor with a Ph.D. in linguistics reviewed and compared the two translations, combining them into a new (first Arabic) version that is conceptually equivalent to the original. The language in this version was kept colloquial and straightforward to ensure accessibility and ease of understanding for Arabic-speaking patients.

The second step, back-translation, is crucial to ensuring that the translated version matches the original. The initial Arabic version from step 1 was translated back into English by two external bicultural experts, both holding Ph.D. degrees in English Language and Literature. To ensure an unbiased assessment of cultural relevance, these experts were unaware of the original questionnaire. The back-translated English version was then compared with the original English questionnaire, and any differences between the translations from steps 1 and 2 were addressed iteratively. This process led to the development of a second Arabic version of the UW-QoL v4, which was then used in the third step.

This is the third step in linguistic validation and testing, involving a focus group. This step aimed to administer the translated questionnaire (second Arabic version) to a sample of respondents to assess whether the translation, including the instructions, items, and response choices, was clear and acceptable. This step ensured that the translation was understood and that the language was simple, appropriate, and accessible to the target population.

The second Arabic version was tested with 10 native Arabic speakers aged 18 to 65; 5 were KHCC staff, and 5 were HNC patients who could read. All interviews were conducted at the Speech-Language Clinic at KHCC in January 2023, and data collection from the focus group took approximately two weeks.

Participants were asked to read and complete the questionnaire during face-to-face group interviews. Each interview lasted approximately 30 to 40 minutes, allowing time for reading and participant comments. Afterward, the interviewer asked whether the participant had difficulty understanding the questions and then checked the participant’s interpretation of each item. If issues arose, the interviewer proposed or tested alternative translations (if anticipated) or asked participants to suggest alternatives. All participants’ comments were reviewed, and the linguistic professor made revisions. The primary concern was question 2, specifically the statement about appearance, which included an option indicating that the disease disfigured the person’s appearance. All participants disliked this translation, prompting a revision. The wording was adjusted to preserve the conceptual meaning while minimizing its impact on the patient’s self-esteem.

Additionally, the focus group provided recommendations for minor grammatical adjustments, such as changing words from plural to singular or vice versa. For questions 10 and 12, additional words were added to the Arabic translation to better align with the original draft (see Appendices A and B for details; changes are highlighted in red). At the conclusion of this step, a two-week proofreading process was conducted on the final Arabic UW-QoL V4 draft. A final linguistic version was prepared for statistical validation, as illustrated in Appendix C.

### Second phase: statistical validation and reliability

In this phase, the finalized Arabic UW-QoL v4 was administered to a larger sample following completion of linguistic validation. Phase one participants were not included in phase two psychometric analyses. Consecutive patients in the HNC department at KHCC were recruited between April 2023 and August 2023. Patients were eligible if they were aged 18 years or older, had histologically confirmed HNC, and were able to speak and read Arabic. Newly diagnosed patients and patients who had completed treatment were eligible; patients receiving palliative care and those with metastatic disease were not excluded. The sample primarily comprised squamous cell carcinoma cases, with other malignancies represented only minimally. The time since treatment completion varied among participants and was not restricted to a fixed interval.

Eligible patients were invited to participate in the study and provided informed consent; the study was approved by the KHCC Ethics Committee. Demographic and clinical data, including age, sex, tumor site, TNM stage, histologic tumor type, and treatment plan, were recorded as characteristics of the study sample (see Appendix D).

### Instruments and procedures

The current version 4 of the UW-QoL questionnaire comprised 12 single-item domains, each with 3 to 6 response options, scaled from 0 (worst) to 100 (best) according to the response hierarchy. Each domain was measured by a single item, with the recall period covering the previous 7 days. The domains included pain, appearance, activity, recreation, swallowing, chewing, speech, shoulder, taste, saliva, mood, and anxiety. In addition, patients were asked to select up to three domains that had been most significant to them.

The questionnaire also includes three global questions: one comparing how patients feel now with how they felt before their cancer diagnosis, one assessing HR-QoL, and one evaluating overall quality of life. For the overall QoL question, patients are instructed to consider their physical and mental health, as well as other factors such as family, friends, spirituality, and leisure activities, that contribute to their life satisfaction. The questionnaire focuses on the patient’s health and quality of life over the past seven days (10).

Patients were asked to complete two sets of questionnaires. The first set was administered during their visit to the SLP clinic, and the retest set was completed 10 days to no more than two weeks after the first administration. This interval was chosen to measure test-retest reliability while reducing the likelihood that patients would recall their first responses (10). The Arabic UW-QoL v4 was used as the primary tool for this study.

### Questionnaire score and statistical analysis

All analyses were executed in R 4.3.2 (R Core Team, Vienna) using *readxl*, *janitor*, *dplyr*, *psych*, and *irr*. The collected data and scripts are in the supplementary material (Appendices D, E).

#### Data preparation

Excel files were imported, headers were harmonized to standardized names, and item scores were coerced into numeric variables. Duplicate IDs were collapsed to the earliest complete record. Rows with more than 30% missing UW-QoL items were excluded, and the remaining missing cells were handled using pairwise deletion when the proportion of missing data was low. For phase two, 151 eligible patients were approached, 118 consented, and 98 were included in the final analytic baseline sample after application of data-cleaning and missing-data rules. The matched test-retest sample also included 98 baseline-retest records.

#### Scoring

The 12 UW-QoL v4 domains were scored 0 – 100 (higher = better QoL) and summed (0 – 1200). In accordance with the developer’s manual, physical (10 items) and socio-emotional (2 items) subtotals were also calculated (5).

#### Descriptive statistics & interpretability

Item means ± SD, medians, and ranges were reported. Floor/ceiling effects were defined as percentages scoring 0 or 100; >15% was judged problematic (12). To facilitate clinical interpretation, the standard error of Measurement (SEM = SD·√1–ICC) and minimal detectable change at 95% confidence (MDC_95_ = 1.96·√2·SEM) were computed (13).

#### Reliability

Internal consistency was evaluated using Cronbach’s alpha for the 10-item physical-function subscale and for the total 12-item scale. Because the socio-emotional subscale comprises only two items, its reliability was estimated using the Spearman-Brown coefficient. Values >=0.70 were deemed acceptable; values >=0.90 were interpreted cautiously for group-level use (12, 13).

Test-retest reliability was assessed using a two-way random-effects, absolute-agreement, single-measure ICC(A,1) with 95% confidence intervals for the total score and domain scores. ICC thresholds followed Koo and Li (2016): <0.50 poor, 0.50-0.75 moderate, 0.75-0.90 good, and >0.90 excellent (13).

#### Structural validity

Dimensionality was assessed using exploratory principal component analysis (PCA) of polychoric correlations with varimax rotation. Eigenvalues >1, scree plot inflection, and parallel analysis guided component retention (12). Confirmatory factor analysis was not performed in this initial validation sample; therefore, structural validity is reported as exploratory PCA-based evidence.

#### Construct validity (hypothesis testing)

Associations between UW-QoL domain scores and corresponding patient-identified problems were examined as preliminary construct validity evidence. Because no external disease-specific QoL comparator was administered, these analyses should not be interpreted as a comprehensive assessment of convergent validity. The Issues-Test was selected as a patient-centered measure to capture self-identified quality-of-life concerns.

Known-groups validity was assessed by comparing total UW-QoL scores between patients with early primary tumor stage (T1-2) and those with advanced primary tumor stage (T3-4), as defined by the composite TNM classification. Shapiro-Wilk tests were used to assess normality within each group. Because distributional assumptions were not met in at least one group, the Mann-Whitney U test was used. The mean difference, p-value, and rank-biserial effect size were reported. We hypothesized that QoL would be higher among patients with an early-stage primary tumor.

#### Measurement error & sensitivity

SEM and MDC_95_ were derived from the total-score ICC to quantify the smallest change beyond random error. Responsiveness could not be evaluated because no post-treatment follow-up data were available; this is planned for a future longitudinal study.

#### Significance level

A two-tailed α = 0.05 was applied throughout; 95% confidence intervals accompany all key estimates.

This analytic strategy was informed by COSMIN recommendations and provides an initial evaluation of selected psychometric properties of the Arabic UW-QoL v4, including reliability, structural validity, measurement error, and selected construct-validity hypotheses (12, 14).

## Results

Of 151 eligible patients approached during the recruitment period, 118 agreed to participate, and 33 declined, yielding an accrual rate of 78.1%. Phase one participants involved in linguistic validation and cognitive debriefing were not included in phase two analyses. After application of data-cleaning and missing-data rules, 98 patients were included in the final analytic baseline sample. Twenty consented participants were excluded from the analytic sample because they lacked analyzable phase two baseline data after data screening. The matched baseline-retest sample included 98 records. Participants included both newly diagnosed and post-treatment cases, representing a range of disease stages with a predominance of advanced disease. The time since treatment completion varied among participants (median = 3.6 months, range = 1.5-5.8 months).

### Descriptive statistics

The analytic cohort was primarily male and middle-aged to older, with a mean age of approximately 59 years. Laryngeal malignancy accounted for more than 85% of non-missing cancer-site data; therefore, the findings primarily apply to this site. Most patients had advanced primary tumor stage, and most were former or current smokers, consistent with known HNC risk profiles. **Table 1** shows the recruitment flow and demographic and clinical characteristics. Percentages are based on non-missing data where indicated.

**Table 1:** Recruitment flow and demographic and clinical characteristics of the analytic Phase 2 sample (N = 98).

Variable	Statistic/n (%)
Recruitment flow	Eligible approached = 151 Consented = 118 Declined = 33 Consented but non-analytic after data screening = 20 Final analytic baseline sample = 98 Matched baseline-retest sample = 98
Accrual rate	78.1% (118/151)
Phase 1 linguistic/cognitive sample	10 native Arabic speakers (5 KHCC staff and 5 HNC patients); not included in Phase 2 analyses
Treatment status (consented Phase 2 sample, N = 118; before analytic exclusions)	Newly diagnosed = 26 (22.0%) Completed treatment = 92 (78.0%)
Time since treatment completion among post-treatment patients	Median = 3.6 months Range = 1.5-5.8 months
Age (years)	Mean = 59.4 +/- 10.1 Median = 59 (IQR 53-67) Range = 19-80
Sex	Male = 84 (86.6%) Female = 13 (13.4%) Missing = 1 Percentages based on non-missing n = 97
Cancer site	Laryngeal = 84 (86.6%) Tongue = 3 (3.1%) Oral cavity = 2 (2.1%) Thyroid = 2 (2.1%) Glottic = 1 (1.0%) Oral-lip = 1 (1.0%) Oropharyngeal = 1 (1.0%) Parotid = 1 (1.0%) Salivary gland = 1 (1.0%) Nasopharyngeal = 1 (1.0%) Missing = 1 Percentages based on non-missing n = 97
Tumor T-stage (extracted from composite TNM)	T1 = 8 (8.2%) T2 = 7 (7.2%) T3 = 18 (18.6%) T4 = 64 (66.0%) Missing = 1 Percentages based on non-missing n = 97
Primary tumor stage group	T1-2 = 15 (15.5%) T3-4 = 82 (84.5%) Missing = 1 Percentages based on non-missing n = 97
Smoking status	Former = 59 (60.8%) Current = 28 (28.9%) Never = 10 (10.3%) Missing = 1 Percentages based on non-missing n = 97
Note	Unless otherwise stated, percentages are based on non-missing data; missing values are shown where applicable.

### Validation results

**Figure 1:**
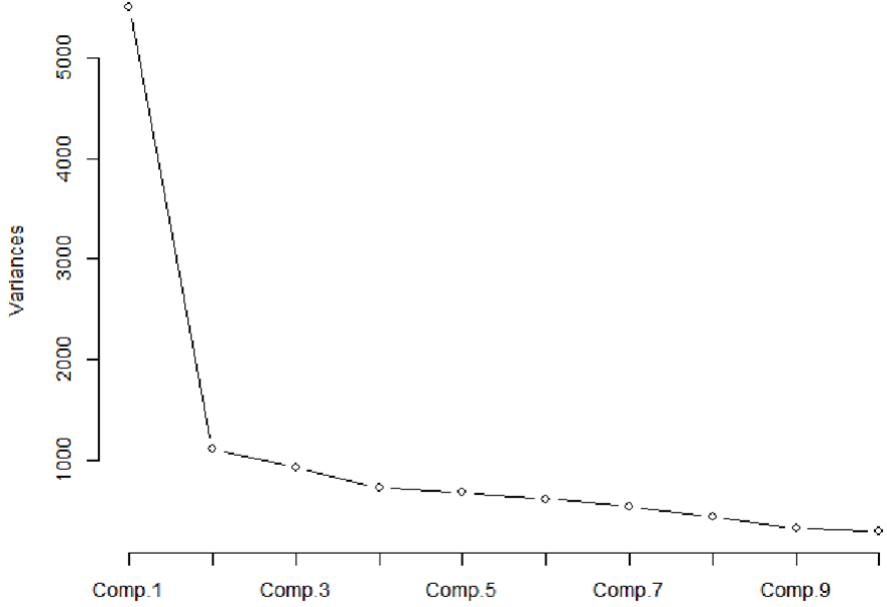
Scree plot from exploratory PCA, showing an inflection after the second component.

Internal consistency was good for the 10-item physical-function subscale (Cronbach’s alpha = 0.86) and for the total 12-item scale (Cronbach’s alpha = 0.89). For the two-item socio-emotional subscale, the Spearman-Brown coefficient was 0.86. Exploratory PCA yielded eigenvalues of 5.67 and 1.06 for the first two components, with all subsequent components below 1; the scree plot showed an inflection after the second component (**Figure 1**), supporting a two-component exploratory solution.

Test-retest reliability over the protocol interval of 10 days to no more than two weeks was excellent for the total score. In 98 matched baseline-retest records, the total-score ICC(A,1) was 0.95. The associated standard error of measurement was 55.6 points on the 0–1200 scale, giving an MDC95 of 154.0 points. Floor effects ranged from 4.1% to 14.3% across domains. Ceiling effects were more prominent, ranging from 26.5% to 50.0%. Associations between domain scores and patient-identified problems were moderate and negative, in the expected direction (r = -0.45 to -0.30). Known-groups testing compared total QoL between early primary tumor stage (T1-2) and advanced primary tumor stage (T3-4). Shapiro-Wilk testing indicated that the normality assumption was not met in at least one group; therefore, the Mann-Whitney U test was used. Early-stage patients had higher total scores than advanced-stage patients (mean difference = 139 points; Mann-Whitney U test, p = 0.0025; rank-biserial correlation = 0.49). This finding for known groups should be interpreted with caution because the early-stage group was small relative to the advanced-stage group. **Table 2** summarizes the psychometric properties of the Arabic UW-QoL v4.

**Table 2:** Arabic version of UW-QoL v4 psychometric properties.

Property	Statistic (criterion)	Result	Interpretation
Internal consistency	Cronbach’s alpha, Physical (Q1-Q10)	0.86	Good internal consistency (>0.70).
Internal consistency	Cronbach’s alpha, Total scale (Q1-Q12)	0.89	Good overall reliability; below the level usually considered excessive for group-level use.
Two-item reliability	Spearman-Brown, socio-emotional (Q11-Q12)	0.86	Appropriate reliability estimate for a two-item subscale.
Structural validity	Exploratory PCA eigenvalues	5.67 and 1.06; all subsequent eigenvalues <1	Exploratory PCA supported a two-component solution; CFA was not conducted.
Structural validity	Component retention	Kaiser criterion and scree-plot inflection supported two components; parallel analysis reviewed	Findings should be interpreted as exploratory structural evidence.
Test-retest reliability	Matched sample and total-score ICC(A,1)	n = 98; ICC(A,1) = 0.95	Excellent agreement using a two-way random-effects, absolute-agreement, single-measure model.
Measurement error	SEM	55.6 points on the 0–1200 scale	Lower values indicate less random measurement error.
Measurement error	MDC95	154.0 points (approximately 12.8% of the total scale)	A change of at least 154 points may exceed random measurement error.
Floor/ceiling effects	% at 0/100	Floor 4.1-14.3%; ceiling 26.5-50.0%	Floor effects were minimal; ceiling effects indicate limited ability to detect improvement in higher-functioning patients.
Preliminary construct validity	Point-biserial correlations between domain scores and patient-identified problems	r = -0.45 to -0.30	Moderate negative associations in the expected direction; interpreted cautiously because no external validated comparator was administered.
Known-groups validity	Early T1–2 vs advanced T3–4 primary tumor stage	Mean difference = 139 points; Mann-Whitney U test p = 0.0025; rank-biserial correlation = 0.49	Better QoL in early primary tumor stage as hypothesized; interpretation limited by imbalance between groups and one missing stage value.

## Discussion

This study translated, culturally adapted, and psychometrically evaluated the Arabic version of the UW-QoL v4 for use among Jordanian patients with HNC. The findings support the conclusion that the Arabic UW-QoL v4 has good internal consistency and excellent test-retest reliability, along with initial evidence of exploratory structural validity and selected construct-validity hypotheses.

The Arabic version demonstrated good internal consistency, with Cronbach’s alpha = 0.86 for the physical-function subscale and 0.89 for the total scale. For the two-item socio-emotional subscale, the Spearman-Brown coefficient was 0.86, which is more appropriate for a two-item scale than reporting Cronbach’s alpha alone. Exploratory PCA supported a two-component solution corresponding broadly to physical and socio-emotional dimensions; however, this should be interpreted as exploratory structural evidence rather than confirmatory factor-analytic evidence. Test-retest reliability was excellent, with a total-score ICC(A,1) of 0.95.

The cultural adaptation process was critical in ensuring the translated tool’s linguistic clarity and cultural relevance. The process followed internationally accepted guidelines, including forward and back translation by bilingual experts, expert panel review, and focus group testing with native Arabic-speaking patients. This approach enabled refinement of problematic items, particularly the wording of the appearance-related question, which was perceived as emotionally distressing and was revised accordingly. Similar psychometric findings were reported in the Sudanese Arabic adaptation of the FACT-H&N (α ≈ 0.85–0.90) (16, 17). This supports our internal consistency results and highlights the importance of culturally sensitive adaptation in Arabic-speaking populations (15). These steps helped ensure that the final Arabic version was linguistically accurate, culturally appropriate, and patient-centered.

Our psychometric evaluation was guided by COSMIN-informed recommendations; however, not all measurement properties were assessed, and the findings should be interpreted as an initial validation. Associations between domain scores and corresponding patient-identified problems provided preliminary construct-validity evidence in the expected direction, but the absence of an external validated comparator limits interpretation as full convergent validity. Known-groups validity testing further supported the tool’s sensitivity to clinical variation, with higher total QoL scores among patients with early primary tumor stage than those with advanced primary tumor stage (Mann-Whitney U test p = 0.0025; rank-biserial correlation = 0.49). Measurement-error analysis indicated an MDC95 of 154.0 points on the 0–1200 scale, which can help guide interpretation of change in future applications.

Despite these strengths, the study had several limitations. The sample was drawn from a single specialized cancer center and was predominantly composed of patients with laryngeal cancer and advanced primary tumor stage, which may limit generalizability to broader Arabic-speaking populations and other HNC sub-sites. The known-groups analysis was also imbalanced, with a much smaller early-stage group than the advanced-stage group; therefore, the statistically significant group difference should be interpreted cautiously. Ceiling effects were observed across all domains, ranging from 26.5% to 50.0%, potentially limiting sensitivity to improvement among higher-functioning patients. Detailed subgroup analyses by treatment status and time since treatment completion were not available for all analyses, which may limit the interpretation of group-specific differences. While test-retest reliability was established, responsiveness to clinical change could not be assessed because longitudinal follow-up data were not available. Future research should include longitudinal studies to evaluate responsiveness, incorporate additional validated instruments to strengthen convergent and divergent validity, and assess the tool across a broader range of clinical subgroups.

## Conclusion

The Arabic version of the UW-QoL v4 was successfully translated and culturally adapted for use among Jordanian HNC patients. In this initial psychometric evaluation, the instrument demonstrated good internal consistency, excellent test-retest reliability, acceptable measurement error, and preliminary evidence of exploratory structural and construct validity. The Arabic UW-QoL v4 can be used in clinical and research settings to support outcome evaluation, treatment planning, and quality-of-life monitoring in comparable Jordanian HNC populations. Further studies using external validated comparators, broader known-groups hypotheses, and longitudinal designs are needed to complete the psychometric evaluation and assess responsiveness over time.

## Data Availability

The original contributions presented in the study are included in the article/supplementary material, further inquiries can be directed to the corresponding author/s.
